# Genome-Wide Identification of *SWEET* Gene Family and Integrative Transcriptomic–Metabolomic Analysis Reveal Sugar Transport-Mediated Chilling Responses in Sesame (*Sesamum indicum* L.)

**DOI:** 10.3390/cimb48030312

**Published:** 2026-03-14

**Authors:** Pan Zeng, Yunyan Zhao, Junchao Liang, Xiaowen Yan, Zhiqi Wang, Jian Sun

**Affiliations:** Jiangxi Province Key Laboratory of Oilcrops Genetic Improvement, Crops Research Institute of Jiangxi Academy of Agricultural Sciences, Nanchang Branch of National Center of Oilcrops Improvement, Nanchang 330200, China; 15271750715@163.com (P.Z.);

**Keywords:** sesame, SWEET transporter, cold stress

## Abstract

Sesame is a thermophilic oilseed crop that is vulnerable to low-temperature stress. SWEET sugar transporters are important for sugar allocation, but their roles in sesame cold responses remain poorly understood. In this study, 24 *SWEET* genes were identified in the sesame genome and classified into six conserved groups with high structural conservation and limited duplication. Comparative transcriptomic and metabolomic analyses of cold-tolerant and cold-sensitive sesame accessions under chilling stress revealed distinct *SiSWEET* expression patterns and contrasting soluble sugar accumulation. Several *SiSWEET* genes showed significant correlations with glucose, fructose, and sucrose contents. These results suggest that SWEET-mediated sugar transport is involved in sesame chilling responses and provide candidate genes for improving cold tolerance.

## 1. Introduction

*Sesamum indicum* L. (sesame), one of the oldest oilseed crops, is valued for its high seed oil content (approximately 50–60%), premium protein quality, and abundant antioxidants such as sesamin and sesamolin [1]. Originating in Africa and widely cultivated in tropical and subtropical regions, sesame serves as a vital economic crop in many developing countries [2]. However, sesame exhibits high sensitivity to low-temperature stress, particularly during the seedling stage, where chilling induces growth retardation, membrane damage, and substantial yield losses, thereby constraining its expansion into temperate zones [3].

Cold stress, including chilling (0–20 °C) and freezing (<0 °C), severely constrains plant growth and productivity by disrupting membrane integrity, metabolic homeostasis, and cellular redox balance. At the physiological level, low temperature reduces membrane fluidity and induces phase transitions of lipid bilayers, leading to electrolyte leakage and impaired transport processes. Cold-tolerant plants often increase the proportion of unsaturated fatty acids to maintain membrane stability and functionality under low temperatures [4]. In parallel, the accumulation of compatible solutes such as soluble sugars (glucose, fructose, sucrose), proline, and other osmolytes contributes to osmotic adjustment, membrane stabilization, and cryoprotection [5]. Cold exposure also enhances reactive oxygen species (ROS) production due to disturbances in chloroplast and mitochondrial electron transport chains, necessitating activation of antioxidant defense systems, including superoxide dismutase, catalase, peroxidases, and non-enzymatic antioxidants to mitigate oxidative damage [6]. Collectively, membrane remodeling, osmotic regulation, and antioxidant defense control constitute an integrated network that underpins plant cold tolerance, providing a physiological and molecular framework for investigating the role of sugar transporters in low-temperature adaptation. These sugars also participate in signaling pathways and antioxidant defense mechanisms [7]. The transport and partitioning of sugars represent critical steps in regulating their accumulation, with the Sugars Will Eventually be Exported Transporters (*SWEET*) gene family encoding key uniporters that facilitate bidirectional sugar flux across membranes [8].

SWEET proteins mediate sucrose and hexose transport, contributing to phloem loading/unloading, seed filling, nectar secretion, and responses to abiotic stresses [9]. In several species, *SWEET* members enhance cold tolerance by modulating sugar allocation; for instance, overexpression of *AtSWEET16* [10] or *AtSWEET17* [11] in Arabidopsis elevates soluble sugar levels and freezing resistance. Additionally, disruption of *AtSWEET11* and *AtSWEET12* in Arabidopsis affects freezing tolerance by impairing vascular sugar transport under cold conditions [12]. Similar roles have been reported in other plants: overexpression of *CsSWEET16* [13] or *CsSWEET17* [14] in tea (*Camellia sinensis*) improves cold tolerance through altered sugar transport and accumulation. In *Hemerocallis fulva*, *HfSWEET17* acts as a positive regulator of cold stress response [15]; and in *Prunus mume*, multiple *PmSWEET* genes respond to low-temperature signals, potentially aiding osmotic balance via sugar redistribution [16].

In the present study, a comprehensive genome-wide identification of the *SiSWEET* gene family was first conducted, revealing 24 members in the sesame genome. Phylogenetic analysis, gene structure characterization, conserved motif identification, cis-regulatory element prediction in promoter regions, intra-genomic synteny analysis within sesame, and inter-genomic synteny comparison with Arabidopsis thaliana were performed to elucidate the evolutionary relationships, structural diversity, and potential regulatory mechanisms of *SiSWEET* genes. Subsequently, transcriptomic and metabolomic profiling were employed to investigate *SiSWEET* gene expression patterns and soluble sugar dynamics in cold-tolerant (RS) and cold-sensitive (SS) sesame accessions under low-temperature treatment (0, 12, and 24 h). This integrated approach aims to uncover the roles of *SiSWEET* genes in sesame cold stress responses and provide candidate genes and theoretical foundations for molecular breeding to enhance chilling tolerance in this important oilseed crop.

## 2. Materials and Methods

### 2.1. Plant Materials and Low-Temperature Treatment

In our previous studies [17], 324 Jiangxi province local sesame germplasm resources were subjected to low-temperature stress, from which the cold-tolerant ZH7 (RS) and cold-sensitive ZH17 (SS) were identified. In this study, ZH7 (RS) and ZH17 (SS) were selected as materials. Seeds were surface-sterilized and germinated on moist filter paper at 28 °C in the dark for 6 days. Uniform seedlings were then transferred to a growth chamber under controlled conditions (28 °C day/28 °C night, 24 h dark photoperiod, 60–70% relative humidity). According to previous research findings [18], for low-temperature stress treatment, seedlings were subjected to chilling at 18 °C (continuous light and humidity maintained), while control seedlings remained at 28 °C. Aerial parts (shoots) were sampled at 0 h (pre-treatment), 12 h, and 24 h after treatment initiation. Samples were immediately frozen in liquid nitrogen and stored at −80 °C until further analysis. Three biological replicates were prepared for each time point and treatment.

**Figure 1:**
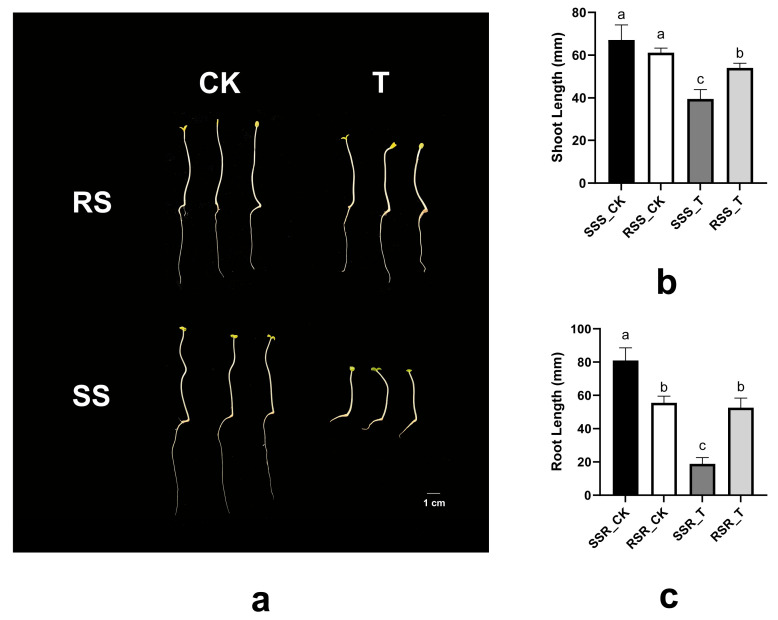
Phenotypic comparison of RS and SS sesame seedlings under chilling treatment (seeds germinated for 6 d. (**a**): Representative images under CK and T conditions; (**b**): shoot length; (**c**): root length. Different lowercase letters indicate significant differences among groups.

To further validate the contrasting cold tolerance of the two genotypes, phenotypic performance under chilling stress was evaluated. Representative images of RS and SS seedlings were recorded during treatment. Shoot length and root length were quantified based on digital images using Image J v1.8.0. Prior to measurement, images were calibrated using a reference scale included in each photograph. RS showed significantly less reduction in shoot and root elongation compared with SS under chilling conditions, supporting its classification as a cold-tolerant genotype (Figure 1). These phenotypic data provide evidence for the subsequent comparative transcriptomic and metabolomic analyses.

### 2.2. Genome-Wide Identification of SiSWEET Genes

The whole-genome sequence, coding sequences (CDS), protein sequences, and annotation files of sesame cultivar Zhongzhi No. 13 were downloaded from NCBI (assembly accession: APMJ00000000). *Arabidopsis thaliana* SWEET protein sequences and genome data were retrieved from TAIR database [19].

To identify potential SiSWEET members, the hidden Markov model (HMM) profile of the SWEET domain (PF03083) was obtained from the Pfam database [20] and used for genome-wide searching with HMMER v3.3.1 (e-value threshold < 1 × 10^−5^) [21]. Candidate proteins were further validated using the NCBI Conserved Domain Database (CDD) tool [22] to confirm the presence of complete MtN3/saliva domains. Only proteins with intact SWEET domains were retained, resulting in 24 SiSWEET members.

### 2.3. Physicochemical Properties and Chromosomal Localization

Physicochemical properties of SiSWEET proteins, including molecular weight, isoelectric point (pI), instability index, and grand average of hydropathicity (GRAVY), were predicted using the ProtParam tool on the ExPASy server [23]. Chromosomal locations of *SiSWEET* genes were extracted from genome annotation files and visualized using TBtools v1.0987663 [24].

### 2.4. Phylogenetic Analysis

Multiple sequence alignment of SiSWEET proteins, along with Arabidopsis AtSWEET proteins, was performed using ClustalW in MEGA 7.0 [25]. A phylogenetic tree was constructed using the Neighbor-Joining (NJ) method with 1000 bootstrap replicates. The tree was visualized and annotated using the Interactive Tree Of Life (iTOL) online platform [26].

### 2.5. Gene Structure, Conserved Motifs, and Promoter Analysis

Coding sequences (CDS), untranslated regions (UTRs), and exon-intron structures of SiSWEET genes were retrieved from genome annotations and confirmed via the NCBI-CDD and Pfam databases. Conserved protein motifs were identified using the MEME Suite (v5.5.1) [27] with default parameters (maximum 10 motifs). For promoter analysis, 2000 bp upstream sequences from the translation start sites (ATG) of each *SiSWEET* gene were extracted and submitted to the PlantCARE database to identify cis-regulatory elements [28]. Gene structures, motif distributions, and promoter elements were visualized using TBtools.

### 2.6. Synteny and Ka/Ks Analysis

Intra-genomic synteny within sesame and inter-genomic synteny between sesame and Arabidopsis were analyzed using MCScanX [29], with the results visualized in TBtools.

### 2.7. Transcriptomic and Metabolomic Analysis

Transcriptomic and untargeted metabolomic analyses were outsourced to Bena Biotechnology (Wuhan, China). For transcriptomics, total RNA was extracted from shoot samples using TRIzol reagent (15596026, Invitrogen, Carlsbad, CA, USA), and RNA integrity was assessed using a NanoDrop spectrophotometer (NanoDrop Technologies, Wilmington, DE, USA) and an Agilent 2100 Bioanalyzer (76336, Waldbronn, Germany; RIN ≥ 7.0). Sequencing libraries were prepared with the NEBNext Ultra RNA Library Prep Kit for Illumina (New England Biolabs, Massachusetts, USA), followed by paired-end sequencing (150 bp reads) on an Illumina NovaSeq 6000 platform. Raw reads were quality-filtered using FastQC and trimmed with Trimmomatic v0.39 [30] to remove adapters and low-quality bases (Phred score < 20). Clean reads were assembled de novo using Trinity v2.8.5 [30], and transcript quantification was performed with RSEM v1.3.3 [31]. Differential gene expression analysis was conducted using DESeq2 v1.30.1 [32] with genes considered differentially expressed at |fold change| ≥ 2 and adjusted *p*-value < 0.05. Gene Ontology (GO) enrichment analyses were performed using clusterProfiler v4.0 in R [33]. Alternative splicing events were detected using rMATS v4.1.0 [34].

For untargeted metabolomics, shoot samples (approximately 100 mg fresh weight) were homogenized in liquid nitrogen and extracted with ice-cold methanol/water (3:1, *v*/*v*) containing internal standards. Extracts were analyzed by ultra-high-performance liquid chromatography coupled with high-resolution mass spectrometry (UHPLC-HRMS) on a Vanquish UHPLC system (Thermo Fisher Scientific, Germany) interfaced with a Q Exactive HFX Orbitrap mass spectrometer (Thermo Fisher Scientific, Germany). Chromatographic separation was achieved on a Waters ACQUITY UPLC HSS T3 C18 column (1.8 µm, 2.1 mm × 100 mm) using a binary gradient of 0.1% formic acid in water (mobile phase A) and 0.1% formic acid in acetonitrile (mobile phase B). Full-scan MS data were acquired in both positive (ESI+) and negative (ESI−) ionization modes with a scan range of *m*/*z* 70–1050. Raw data preprocessing, including peak detection, alignment, and normalization, was performed using Compound Discoverer v3.1 (Thermo Fisher Scientific). Metabolite annotation was conducted by matching accurate mass, retention time, and MS/MS spectra against in-house databases, HMDB, METLIN, and KEGG. Quality control (QC) samples were injected periodically to monitor instrument stability, and system performance was evaluated through total ion chromatograms (TICs) and base peak chromatograms (BPCs). Differential metabolites were identified based on variable importance in projection (VIP) >1.0, and *p*-value < 0.05 (Student’s t-test). Pathway enrichment analysis of differential metabolites was performed using MetaboAnalyst v5.0 with the Kyoto Encyclopedia of Genes and Genomes (KEGG) database [35].

Spearman correlation analysis was performed to assess the relationships between SiSWEET gene expression levels and Fsugar contents across RS and SS samples (*n* = 6). Due to the limited sample size and non-normal distribution of metabolite data, Spearman’s rank correlation was applied. Correlations with |r| ≥ 0.7 and *p* < 0.05 were considered significant.

### 2.8. Quantitative RT–PCR

We selected 10 genes for qRT--PCR assays. The total RNA of the stem was extracted using an RNAprep pure Plant Kit (Tiangen, DP441, Beijing, China) following the manufacturer’s instructions. RNA was treated with RNase-free DNase I to remove genomic DNA. Firststrand cDNA was synthesized using a RevertAid First Strand cDNA Synthesis Kit (Thermo Scientific, Waltham, MA, USA). Gen-specific primer sequences and detailed information are given in Table S3. qRT-PCR reactions were conducted using SYBR Green Realtime PCR Master Mix (Toyobo, Osaka, Japan) with a CFX96 Touch Real-Time PCR Detection System (Bio-Rad, Hercules, CA, USA). The thermal cycler was performed as follows: 1 cycle of 95 °C 30 s; followed by 40 cycles of 95 °C for 10 s, 60 °C for 30 s, and 72 °C for 30 s. The 2^−∆∆Ct^ method was used to analyze the results with CFX Manager v3.1 (Bio-Rad, Hercules, CA, USA). Three biological replicates (with three technical replicates for each biological replicate) were analyzed for each sample.

## 3. Results

### 3.1. Identification of SiSWEET Gene Family Members

**Figure 2:**
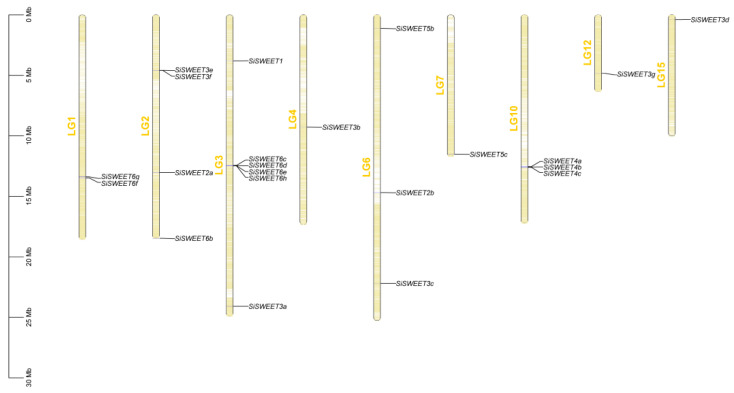
Chromosomal location information of *SiSWEETs*.

A total of 24 SiSWEET protein-coding genes were identified in the sesame genome through a combined approach using HMMER searches, with further validation and domain integrity check performed using the NCBI-CDD tool. Proteins lacking complete conserved domains were excluded. The remaining 24 SiSWEET genes were designated as SiSWEET1 to SiSWEET6h according to their phylogenetic clustering in the evolutionary tree (primarily based on clade affiliation) and their physical chromosomal positions (Table 1). Among these genes, 22 were successfully mapped to specific chromosomes or linkage groups (LGs), while two (*SiSWEET5a*, *SiSWEET6a*) were located on the contigs. Chromosomal distribution analysis showed that SiSWEET genes were unevenly distributed across the *S. indicum* genome (Figure 2).

The physicochemical properties of the 24 SiSWEET proteins were analyzed, including isoelectric point (pI), molecular weight, instability index, aliphatic index, and grand average of hydropathicity (GRAVY), as summarized in Table 1. The predicted molecular weights (MW) of SiSWEET proteins ranged from 17.68 kDa (SiSWEET6h) to 33.44 kDa (SiSWEET6c). The theoretical pI values varied from 5.87 (SiSWEET6c) to 9.76 (SiSWEET6g), indicating that the SiSWEET proteins include both acidic and basic types.

The instability index, which reflects protein stability in vitro, ranged from 27.45 (SiSWEET3d) to 57.34 (SiSWEET4b). According to the standard threshold (instability index > 40 indicates potential instability), 19 SiSWEET proteins were predicted to be unstable, while the remaining were considered stable. The aliphatic index values varied from 108.43 (SiSWEET2a) to 128.22 (SiSWEET1), suggesting moderate aliphatic amino acid content among the proteins.

The GRAVY values of SiSWEET proteins ranged from 0.283 to 1.187, with SiSWEET6h showing the highest value. These data describe the overall physicochemical properties of the identified proteins.

### 3.2. Phylogenetic Analysis of SiSWEET Proteins

**Figure 3:**
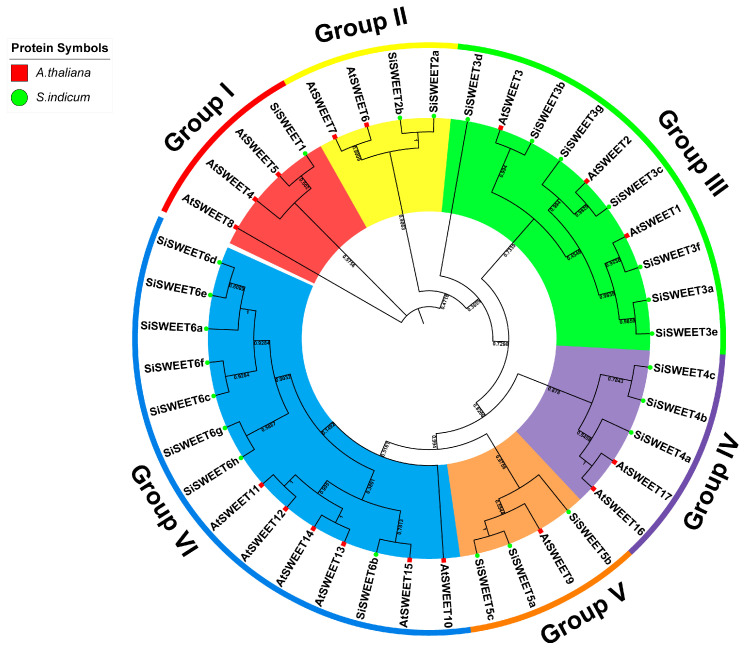
Phylogenetic tree of SWEET proteins in *A. thaliana*, *S. indicum*.

A Neighbor-Joining phylogenetic tree was constructed using the amino acid sequences of the 24 SiSWEET proteins and 17 AtSWEET proteins from *Arabidopsis thaliana*, dividing the SiSWEET family into six major groups (Group I–VI) consistent with their nomenclature (Figure 3). Group VI, the largest, comprised eight SiSWEET members (SiSWEET6a–SiSWEET6h) and clustered with AtSWEET10–AtSWEET15, forming several distinct subclusters. Group V included three SiSWEET proteins (SiSWEET5a–SiSWEET5c) grouping closely with AtSWEET9. Group IV contained three SiSWEET proteins (SiSWEET4a–SiSWEET4c) clustering with the vacuolar transporters AtSWEET16/AtSWEET17. Group III consisted of seven SiSWEET proteins (SiSWEET3a–SiSWEET3g) grouping with AtSWEET1–AtSWEET3, showing clear subclustering. Group II included two SiSWEET proteins (SiSWEET2a, SiSWEET2b) clustering with AtSWEET6–AtSWEET8. Group I comprised a single SiSWEET protein (SiSWEET1) associated with AtSWEET4/AtSWEET5. Overall, the tree exhibited robust node support, indicating reliable relationships; the grouping pattern suggests functional specialization, with larger groups (III and VI) likely involved in diverse sugar transport roles, and close paralog clustering points to duplication events driving family expansion in sesame.

### 3.3. Conserved Motifs, Gene Structure, and Promoter Analysis of SiSWEET Genes

**Figure 4:**
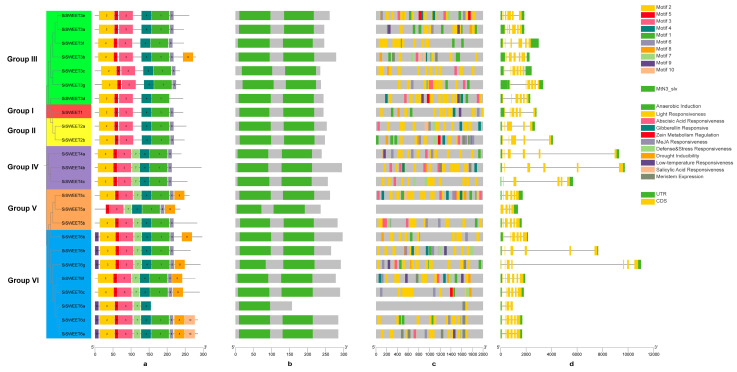
Conserved motifs, gene structure, and promoter analysis of *SiSWEET* genes. (**a**): Conserved motifs of SiSWEET family proteins; (**b**): Pfam structure of SiSWEET family proteins; (**c**): cis-acting elements in the promoters of *SiSWEET* genes; (**d**): the mRNA structure encoded by the *SiSWEETs*.

To further characterize the structural and regulatory features of the 24 SiSWEET genes, conserved motifs, exon-intron organization, and cis-regulatory elements in promoter regions were analyzed (Figure 4). Ten conserved motifs were identified in SiSWEET proteins using the MEME suite, with most proteins containing 6–10 motifs. Motifs 1–6 were widely distributed across all SiSWEET proteins and corresponded to the characteristic MtN3/saliva domains (PF03083), as confirmed by NCBI-CDD analysis, reflecting the conserved transmembrane helices essential for sugar transport function (Figure 4a,b).

Analysis of 2000 bp upstream promoter regions using PlantCARE identified abundant cis-regulatory elements related to light responsiveness and hormone responsiveness. Elements associated with meristem expression and low-temperature response were also prevalent in several promoters. Notably, stress-related elements (drought, anaerobic, MeJA, and defense/stress) were enriched in promoters *SiSWEET* genes, indicating potential roles in environmental adaptation, while light-responsive elements were ubiquitous across all groups, consistent with the involvement of *SWEET* genes in photosynthetic sugar partitioning. These findings suggest that *SiSWEET* expression is finely regulated by developmental, hormonal, and stress signals in sesame (Figure 4c). Due to the sequencing depth limitation, *SiSWEET5a*, which is located on a pseudochromosome, lacks annotation information and does not contain any element. Gene structure analysis indicated that the number of exons among *SiSWEET* genes ranged from 1 to 3 (Figure 4d). Additionally, *SiSWEET* genes harbored four to six untranslated regions (UTRs), located at both the 5′ and 3′ ends.

### 3.4. Synteny Analysis of SiSWEET Genes

**Figure 5:**
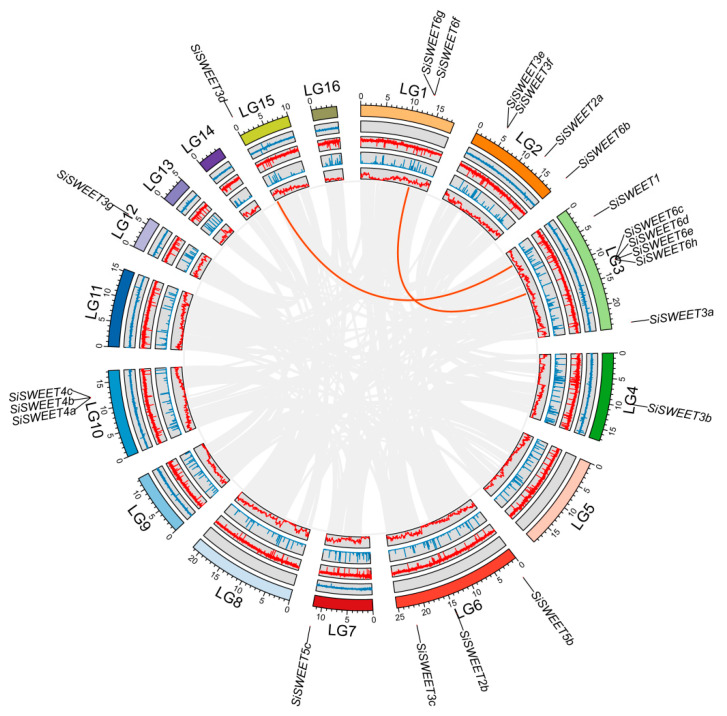
Collinearity of *SiSWEETs.* The circles in the figure, from inside to outside, represent the unknown base N ratio, gene density, GC ratio, GC skew, and chromosome length of the *S. indicum* genome.

**Figure 6:**
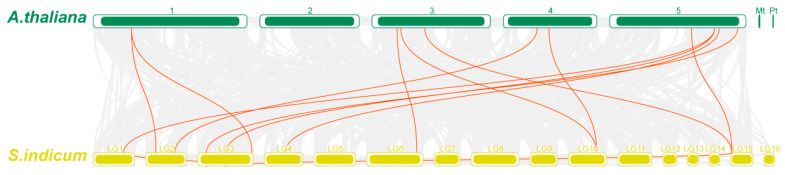
Collinearity of *SWEET* genes in *A. thaliana*, *S. indicum*.

Intra-genomic and inter-genomic synteny analyses were conducted to elucidate the evolutionary history of the *SiSWEET* gene family. Within the sesame genome, only two syntenic gene pairs were identified: *SiSWEET6f*/*SiSWEET6c* (both in Group VI) and *SiSWEET3d*/*SiSWEET1* (Group III and Group I, respectively), indicating that segmental duplication events have contributed minimally to family expansion, with most *SiSWEET* members likely derived from ancient duplications or lineage-specific retention (Figure 5 and Table S1). Comparative synteny with *A. thaliana* revealed 12 orthologous pairs involving 10 *SiSWEET* genes and 9 *AtSWEET* genes (Figure 6 and Table S2). These conserved syntenic blocks reinforce the phylogenetic relationships and orthology across clades, while instances of multiple sesame genes corresponding to single Arabidopsis orthologs suggest limited lineage-specific expansion in sesame following divergence; overall, the results underscore evolutionary conservation of *SWEET* genes among dicots with modest duplication and divergence in the sesame lineage.

### 3.5. Global Transcriptomic and Metabolomic Responses to Low-Temperature Stress

Transcriptomic analysis identified a substantial number of differentially expressed genes (DEGs; |log_2_FC| ≥ 1, adjusted *p*-value < 0.05) in response to low-temperature stress (Table 2). In the cold-tolerant accession (RS), 2186 DEGs were detected at 12 h compared to 0 h (1064 up, 1122 down), decreasing to 793 DEGs at 24 h vs. 0 h (262 up, 531 down), with 543 DEGs between 12 h and 24 h. In the cold-sensitive accession (SS), similar numbers were observed at 12 h vs. 0 h (2142 DEGs; 867 up, 1275 down) and 24 h vs. 0 h (2373 DEGs; 771 up, 1602 down), but only 30 DEGs between 12 h and 24 h. Between accessions, 1145 DEGs were found at 0 h (RS vs. SS), increasing to 1340 at 12 h and decreasing to 732 at 24 h, highlighting dynamic genotypic differences during stress progression. The gene expression results (FPKM) are shown in Table S4.

Untargeted metabolomic profiling detected 563 metabolites across samples, with 15–76 differentially accumulated metabolites (DAMs; VIP > 1.0, *p*-value < 0.05) in various comparisons (Table 3). Time-course changes within accessions were moderate (25–54 DAMs in RS, 15–26 in SS), while inter-accession comparisons revealed more DAMs (58–76), indicating pronounced metabolic divergence between RS and SS under chilling.

To elucidate the functional and metabolic consequences of low-temperature stress, Gene Ontology (GO) enrichment was performed on differentially expressed genes (DEGs) and KEGG pathway enrichment on differentially accumulated metabolites (DAMs).

In the cold-tolerant accession (RS), early chilling triggered strong enrichment of GO terms associated with signaling receptor activity, abscisic acid binding, channel activity, heme binding, peroxidase activity, and hydrogen peroxide catabolic process, reflecting rapid activation of ABA perception, stress signaling, and ROS detoxification mechanisms. Corresponding KEGG pathways prominently included ABC transporters and linoleic acid metabolism, consistent with enhanced transport of osmoprotectants and lipid remodeling for membrane protection. At later stages, GO enrichment shifted toward photosynthetic processes (photosystem II, photosystem I, chlorophyll binding, light harvesting), indicating maintenance or recovery of photosynthetic function, while KEGG highlighted amino acid metabolism (e.g., alanine/aspartate/glutamate), GABAergic synapse, and glutathione metabolism, pointing to amino acid reconfiguration, GABA-mediated signaling, and sustained antioxidant support during acclimation.

In the cold-sensitive accession (SS), early response was dominated by GO terms related to photosynthetic apparatus disruption (photosystem II, photosystem I, light harvesting, chlorophyll binding), suggesting more severe impairment of energy production compared to RS. KEGG enrichments included ABC transporters and arginine/proline metabolism, implying attempts at osmotic adjustment but with limited scope. Later stages featured nicotinate and nicotinamide metabolism in KEGG, potentially linked to redox homeostasis, yet overall metabolic reprogramming appeared weaker.

Inter-accession comparisons consistently enriched GO terms for abscisic acid binding, defense response, and cell wall remodeling (e.g., xyloglucan-related transferase activity), alongside KEGG pathways such as phenylalanine metabolism, phenylpropanoid biosynthesis, and galactose metabolism at both control and stress conditions, underscoring baseline and stress-induced genotypic differences in hormone perception, phenolic secondary metabolism, and sugar handling.

These integrated enrichments indicate that chilling tolerance in RS is supported by coordinated ABA signaling, effective ROS scavenging, photosynthetic resilience, and active solute/osmolyte transport, whereas SS exhibits greater photosynthetic vulnerability and more restricted metabolic adaptation. Detailed GO and KEGG results are provided in Supplementary Tables S5 and S6.

### 3.6. Expression Dynamics of SiSWEET Genes

**Figure 7:**
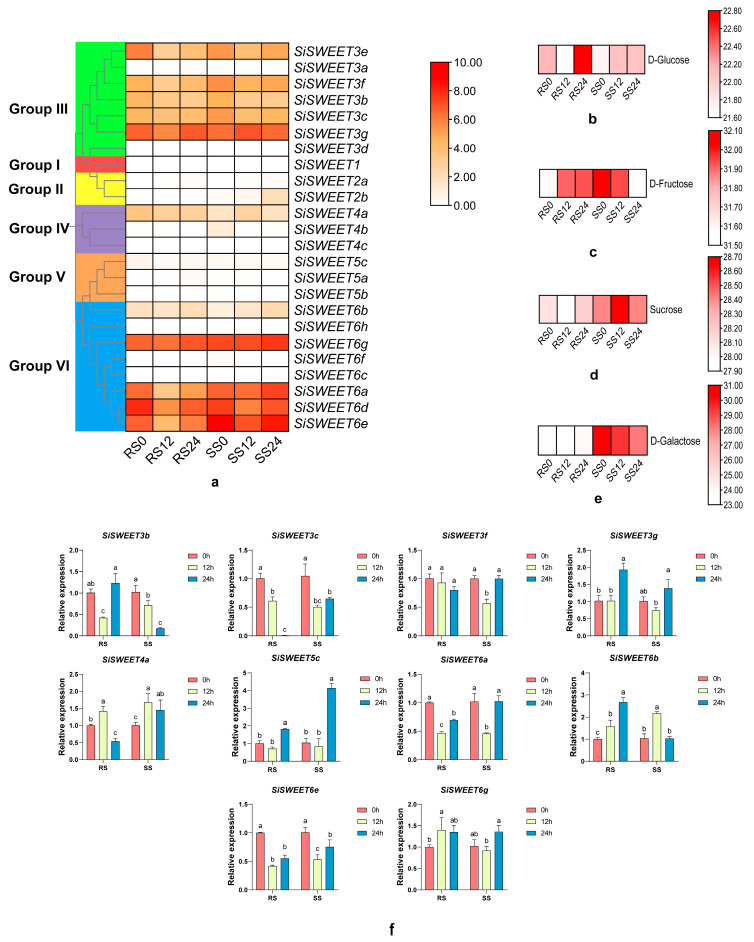
Soluble sugar accumulation patterns. (**a**) Expression profiles of *SiSWEET* genes; (**b**–**e**) Dynamics of soluble sugar accumulation under low-temperature stress; (**f**) qRT-PCR confirmation. Different lowercase letters indicate significant differences among groups.

Among the 24 *SiSWEET* genes, expression levels varied considerably, with several showing negligible or undetectable transcription across samples (Figure 7a, log2 (FPKM + 1) transformation). The highest expression was observed in Group VI, particularly *SiSWEET6a*, *SiSWEET6d*, and *SiSWEET6e*. In RS, these genes were sharply downregulated at 12 h, followed by partial recovery at 24 h. In SS, downregulation was milder or absent, with some genes maintaining or increasing expression at 24 h.

Group III genes exhibited moderate expression, with similar trends: stronger early downregulation and rebound in RS versus sustained suppression or variable recovery in SS. Groups IV and V showed low expression with minor downregulation in RS, while Groups I and II had very low levels with slight induction in SS.

### 3.7. Soluble Sugar Accumulation Patterns

Soluble sugar levels (log_2_ (Relatively abundant + 1) transformation) displayed contrasting dynamics between accessions. In RS, D-glucose decreased initially but strongly rebounded (Figure 7b), D-fructose increased progressively (Figure 7c), sucrose remained stable (Figure 7d), and D-galactose showed no significant change (Figure 7e). In SS, D-glucose rose modestly early without further increase (Figure 7b), D-fructose declined steadily (Figure 7c), sucrose transiently peaked at 12 h (Figure 7d), and D-galactose dropped sharply (Figure 7e). These patterns indicate effective sugar retention and remobilization in RS, contrasting with progressive depletion in SS.

### 3.8. Quantitative RT–PCR and Correlation Between SiSWEET Expression and Soluble Sugar Levels

To further verify the RNA-seq data, ten *SiSWEET* genes were selected for qRT-PCR analysis based on the heatmap patterns, mainly focusing on genes showing relatively distinct and representative differential expression trends under low-temperature stress (Figure 7f). Overall, the qRT-PCR results were largely consistent with the transcriptome data, supporting the reliability of the observed expression patterns. Spearman correlation analysis revealed that several *SiSWEET* genes were significantly associated with sugar accumulation under cold stress (Table S4). For example, *SiSWEET3g* showed a significant positive correlation with glucose content (r = 0.829, *p* < 0.05), whereas *SiSWEET4b* was negatively correlated with glucose (r = −0.886, *p* < 0.05). In addition, sucrose and galactose contents were positively correlated with the expression of *SiSWEET2b*, *SiSWEET6e*, and *SiSWEET6g*, suggesting a potential involvement of these genes in sugar redistribution during cold stress.

## 4. Discussion

The Sugars Will Eventually be Exported Transporters *(SWEET*) family represents a key class of sugar facilitators in plants, mediating passive transport of mono- and disaccharides across membranes and influencing source-sink relationships, seed development, and abiotic stress adaptation [9,36]. Here, we report the first comprehensive genome-wide characterization of the SWEET gene family in sesame (*S*. *indicum* L.), identifying 24 *SiSWEET* members that align with family sizes in other dicots, where expansions often correlate with specialized transport demands rather than widespread duplication [11]. This number exceeds that in *A. thaliana* (17 *AtSWEETs*). Phylogenetic grouping into six clades mirrors the conserved classification across angiosperms, with larger groups (III and VI) likely underpinning diversified transport functions.

Most *SiSWEET* genes exhibit simple exon–intron structures, with one to three exons, a feature commonly observed in membrane transporter genes. Genes with fewer introns are often associated with rapid transcriptional responsiveness, which is advantageous under fluctuating environmental conditions such as sudden temperature drops [37]. This structural simplicity, combined with the high hydrophobicity of SiSWEET proteins, is consistent with their roles as membrane-localized sugar uniporters [8]. Promoter analysis revealed that *SiSWEET* genes harbor abundant cis-acting elements related to light regulation, phytohormone signaling, and abiotic stress responses, including low-temperature elements. Sugars are not only metabolic substrates but also signaling molecules that interact with hormonal and stress-response pathways [38,39]. Therefore, the complex promoter architecture of *SiSWEET* genes suggests that they may serve as regulatory nodes integrating environmental signals with carbohydrate allocation during stress adaptation.

The integrated transcriptomic and metabolomic analyses enabled the prioritization of specific SiSWEET members associated with chilling tolerance rather than attributing the response to the entire gene family. Based on genotype-specific transcriptional dynamics, strong gene–metabolite correlations, and known functional characteristics of SWEET clades, *SiSWEET6e* and *SiSWEET6g* emerged as the strongest candidate genes.

Both genes exhibited pronounced and dynamic expression changes in the cold-tolerant genotype (RS) but not in the cold-sensitive genotype (SS), and their transcript levels were positively correlated with sucrose and galactose accumulation. Members of clade III SWEET are widely reported to mediate sucrose efflux and carbon partitioning [9,36]. Given the central role of sucrose in osmotic adjustment and membrane stabilization during cold stress [4,40], the coordinated regulation of *SiSWEET6e*/*6g* with sucrose dynamics suggests that these genes may contribute to maintaining intracellular carbohydrate balance under chilling conditions.

In addition, *SiSWEET3g* showed a significant positive correlation with glucose accumulation, whereas *SiSWEET4b* displayed a negative association with glucose levels. The contrasting correlation patterns imply distinct roles in hexose redistribution, consistent with previous reports that SWEET family members exhibit substrate specificity and directional transport differences [9]. Notably, the early-stage downregulation of several Group VI members in RS coincided with stabilization of glucose and fructose levels, suggesting that transient suppression of sugar efflux may facilitate intracellular sugar retention during acute chilling exposure. Such sugar retention has been linked to enhanced cold tolerance through osmoprotection and reactive oxygen species buffering [41].

## 5. Conclusions

Taken together, our results indicate that cold tolerance in sesame is closely associated with the ability to dynamically regulate SWEET-mediated sugar transport rather than with constitutively high sugar content. *SiSWEET* genes exhibiting genotype-specific expression patterns and strong correlations with sugar accumulation represent promising candidates for functional characterization. From an applied perspective, these genes may serve as molecular targets for breeding or genetic engineering aimed at enhancing chilling tolerance. Given the limited expansion and high conservation of the *SWEET* family in sesame, fine-tuning the expression of key SiSWEETs may provide an efficient strategy to improve stress resilience without disrupting normal growth and development.

## Figures and Tables

**Table 1:** Gene Information and Protein Physicochemical Characterization of SiSWEETs.

Gene ID	Gene Name	Chr	Start	End	AA	MW (kDA)	pI	II	AI	GRAVY
*gene-LOC105157196*	*SiSWEET1*	LG3	3,798,121	3,800,933	253	28.14	9.65	47.49	128.22	0.789
*gene-LOC105156025*	*SiSWEET2a*	LG2	13,037,724	13,040,413	261	28.46	9.53	27.64	108.43	0.605
*gene-LOC105164502*	*SiSWEET2b*	LG6	14,685,856	14,689,958	246	27.20	9.11	29.6	120.45	0.704
*gene-LOC105159417*	*SiSWEET3a*	LG3	24,087,526	24,089,385	285	31.94	9.42	42.85	113.54	0.689
*gene-LOC105160382*	*SiSWEET3b*	LG4	9,282,977	9,285,248	285	31.94	9.42	42.85	113.54	0.689
*gene-LOC105165480*	*SiSWEET3c*	LG6	22,190,261	22,192,696	279	31.63	8.46	47.9	110.68	0.283
*gene-LOC105177146*	*SiSWEET3d*	LG15	389,191	391,516	256	27.84	9.28	27.45	116.88	0.592
*gene-LOC105179860*	*SiSWEET3e*	LG2	4,580,760	4,582,623	237	26.49	9.49	51.34	123.76	0.947
*gene-LOC105179868*	*SiSWEET3f*	LG2	4,589,612	4,592,602	244	27.18	9.44	28.31	119.75	0.773
*gene-LOC110011242*	*SiSWEET3g*	LG12	4,828,104	4,831,445	236	26.90	9.41	29.93	109.45	0.529
*gene-LOC105172394*	*SiSWEET4a*	LG10	12,538,552	12,547,849	262	29.63	9.31	29.2	116.07	0.677
*gene-LOC105172396*	*SiSWEET4b*	LG10	12,566,543	12,576,285	239	26.56	8.94	57.34	112.09	0.681
*gene-LOC105172791*	*SiSWEET4c*	LG10	12,596,088	12,601,768	295	32.39	9.64	35.1	112.61	0.388
*gene-LOC105155257*	*SiSWEET5a*	-	27	1389	292	33.07	9.2	45.27	124.14	0.643
*gene-LOC105163183*	*SiSWEET5b*	LG6	1,112,361	1,114,018	265	29.23	8.4	29.18	121.77	0.693
*gene-LOC105167215*	*SiSWEET5c*	LG7	11,518,619	11,520,354	235	26.23	8.77	42.49	121.15	0.786
*gene-LOC105155294*	*SiSWEET6a*	-	32	987	278	31.41	8.73	37.14	123.31	0.703
*gene-LOC105156725*	*SiSWEET6b*	LG2	18,464,994	18,467,141	246	27.16	9.4	27.77	112.11	0.626
*gene-LOC105158159*	*SiSWEET6c*	LG3	12,441,206	12,443,027	297	33.44	5.87	41.68	118.08	0.714
*gene-LOC105158492*	*SiSWEET6d*	LG3	12,458,418	12,460,122	244	27.56	8.98	41.49	113.4	0.553
*gene-LOC105158493*	*SiSWEET6e*	LG3	12,470,344	12,472,033	290	32.76	6.89	49.71	126.03	0.743
*gene-LOC105166033*	*SiSWEET6f*	LG1	13,475,992	13,477,921	283	31.92	9.17	43.48	120.85	0.614
*gene-LOC105166923*	*SiSWEET6g*	LG1	13,378,950	13,389,966	248	27.24	9.76	45.58	113.95	0.603
*gene-LOC110011762*	*SiSWEET6h*	LG3	12,475,021	12,482,679	157	17.68	9.26	36.35	125.35	1.187

Chr: Chromosome; AA: Number of Amino Acids; MW: Molecular Weight; pI: Theoretical Isoelectric Point; II: Instability Index; AI: Aliphatic Index; GRAVY: Grand Average of Hydropathicity.

**Table 2:** Number of DEGs in various comparisons.

Comparison	Significant Different Number	Up	Down
RS0 vs. RS12	2186	1064	1122
RS0 vs. RS24	793	262	531
RS12 vs. RS24	543	250	293
SS0 vs. SS12	2142	867	1275
SS0 vs. SS24	2373	771	1602
SS12 vs. SS24	30	9	21
SS0 vs. RS0	1145	436	709
SS12 vs. RS12	1340	719	621
SS24 vs. RS24	732	379	353

**Table 3:** Number of DAMs in various comparisons.

Comparison	Total	Up	Down	Total_DE
RS12 vs. RS0	563	23	31	54
RS24 vs. RS0	563	10	17	27
RS24 vs. RS12	563	18	17	35
SS12 vs. SS0	563	9	17	26
SS24 vs. SS0	563	10	15	25
SS24 vs. SS12	563	8	7	15
RS0 vs. SS0	563	33	43	76
RS12 vs. SS12	563	30	28	58
RS24 vs. SS24	563	26	33	59

## Data Availability

The original contributions presented in this study are included in the article and Supplementary Material. Further inquiries can be directed to the corresponding author.

## Supplementary Materials

The following supporting information can be downloaded at: https://www.mdpi.com/article/10.3390/cimb48030312/s1.
